# Empowerment of Italian general practitioners in depression and suicide prevention: the iFightDepression tool, a game changer of the EAAD-Best project

**DOI:** 10.1186/s12991-024-00506-0

**Published:** 2024-06-26

**Authors:** Nuhara Vargiu, Aurora Belfanti, Michela Roberti, Serena Trentin, Camilla Ferrara, Manuela Tosti, Marco Lazzeri, Giancarlo Giupponi, Andreas Conca

**Affiliations:** Azienda Sanitaria Alto Adige - South Tyrol Health Authority, Bolzano, Italy

**Keywords:** Depression Prevention, Suicide risk, General practitioners (GPs), EAAD, FightDepression, Self-help app, Education

## Abstract

General Practitioners (GPs) play a key role in the early detection and management of depression and in preventing suicide risk. They are often the first healthcare professionals that people in crisis contact. However, their effectiveness can be limited by several barriers, including the lack of specific training and appropriate tools.

The EAAD-Best project aims to fill these gaps through its iFightDepression tool, an online tool designed to support patients, psychologists, psychiatrists, and GPs in managing depression and preventing suicide. This article examines the implementation of the iFightDepression platform in Italy, assessing its impact on the empowerment of GPs in the fight against depression. Through a qualitative and quantitative analysis of the data collected by the project, the ‘unmet need’ of GPs’ in Italy regarding their specific training in mental health is highlighted.

The response of 2,068 Italian GPs in just 7 months after the start of the iFD project is an expression of GPs’ engagement to work against depression and for suicide risk prevention.

## Background

The European Alliance Against Depression (EAAD) is a non-profit organization founded in 2008, dedicated to mitigating depression and reducing suicide risks internationally. The EAAD-Best consortium, which includes Germany, Hungary, Poland, Bulgaria, Greece, Estonia, Spain, Ireland, Belgium, and Italy, emphasizes the adoption of ‘best practices’ in preventing depression and suicide, guided by the ‘4-level intervention’ model [1–3]. It focuses on comprises training healthcare professionals to enhance diagnostic and treatment accessibility, public interventions to reduce stigma, training non-specialized workers (e.g., teachers, police) to recognize depressive symptoms, and providing direct support to affected individuals and their families.

The iFightDepression tool, an initiative within this framework, exemplifies a comprehensive approach to mental health support, facilitating self-guided management of mild to moderate depression using cognitive-behavioral therapy principles [4–6]. This EU co-financed project, running from 2021 to 2024, is a collaboration among multiple European countries, with Italy’s involvement coordinated by the South Tyrol Health Authority as of 2023.

The Italian EAAD-Best team has seized the opportunity provided by the iFD project to support the crucial role of GPs in depression prevention. The correlation between GPs’ training and depression prevention is a topic of great interest in the field of medicine and public health [7, 8]. However, Italy faces challenges such as a declining number of GPs and an aging medical workforce, which could potentially compromise healthcare quality and accessibility. Reports [9–11] predict a significant reduction in GP numbers by 2025. The declining trend in the number of professionals active in general medicine emphasizes the urgent need to concentrate resources and efforts on strengthening the primary healthcare system [12, 13]. The methods employed by the iFightDepression project, led by South Tyrol Health Authority in 2023, involve optimizing the Italian usage of the iFD platform and strategically planning GP engagement and training.

## Main text

Out of the 20 regions of Italy contacted, 12 (60%) responded positively and were interested in the iFD training, with different levels of participation that showed greater openness in some areas in comparison to others, as well as a greater involvement of postgraduate schools.

**Fig. 1:**
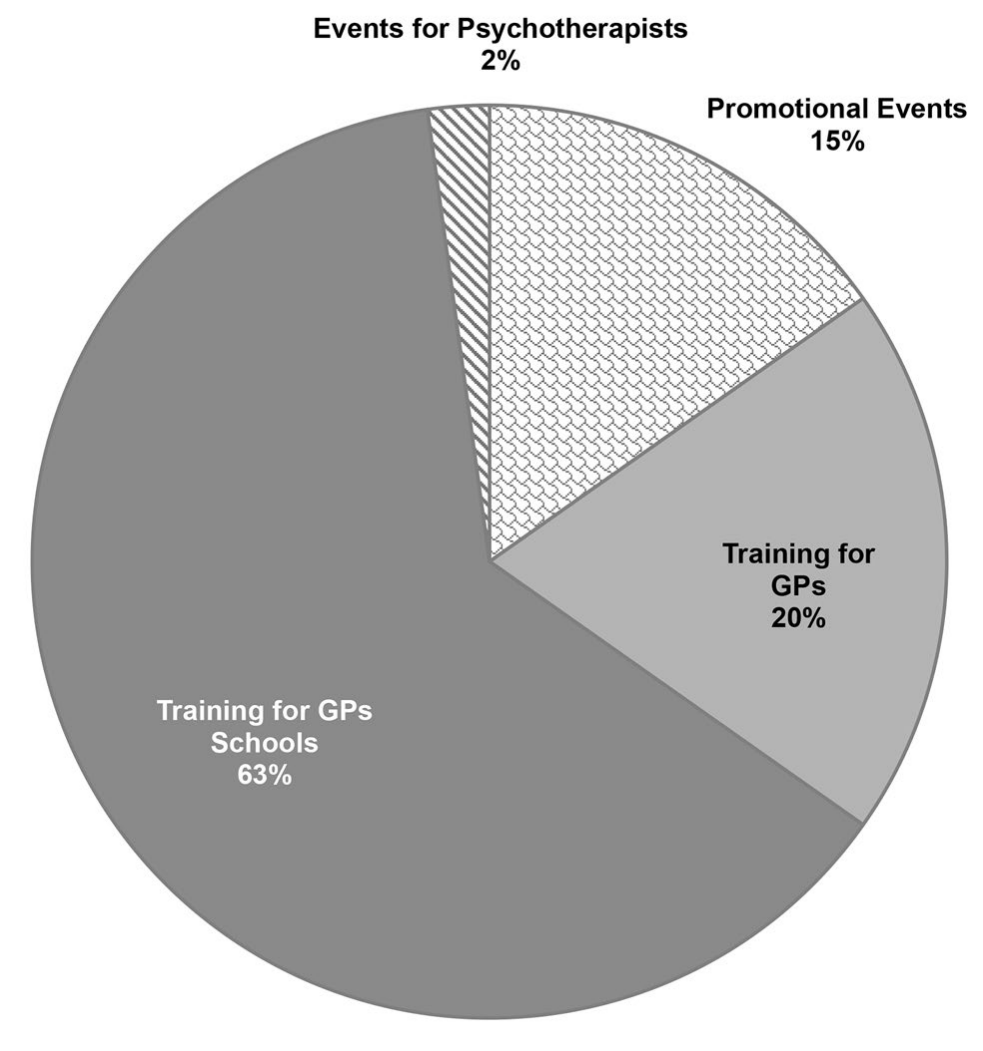
iFightDepression events by public involved (september 2023 – march 2024)

The project included 7 promotional events, including press conferences and initiatives to raise awareness of depression prevention. In addition to these activities, a total of 38 training sessions were organized. These training sessions, which focused on utilizing the iFD tool, depression prevention, and managing suicide risk, were conducted both through university training schools for GPs and directly to already qualified GPs (Fig. 1).

**Fig. 2:**
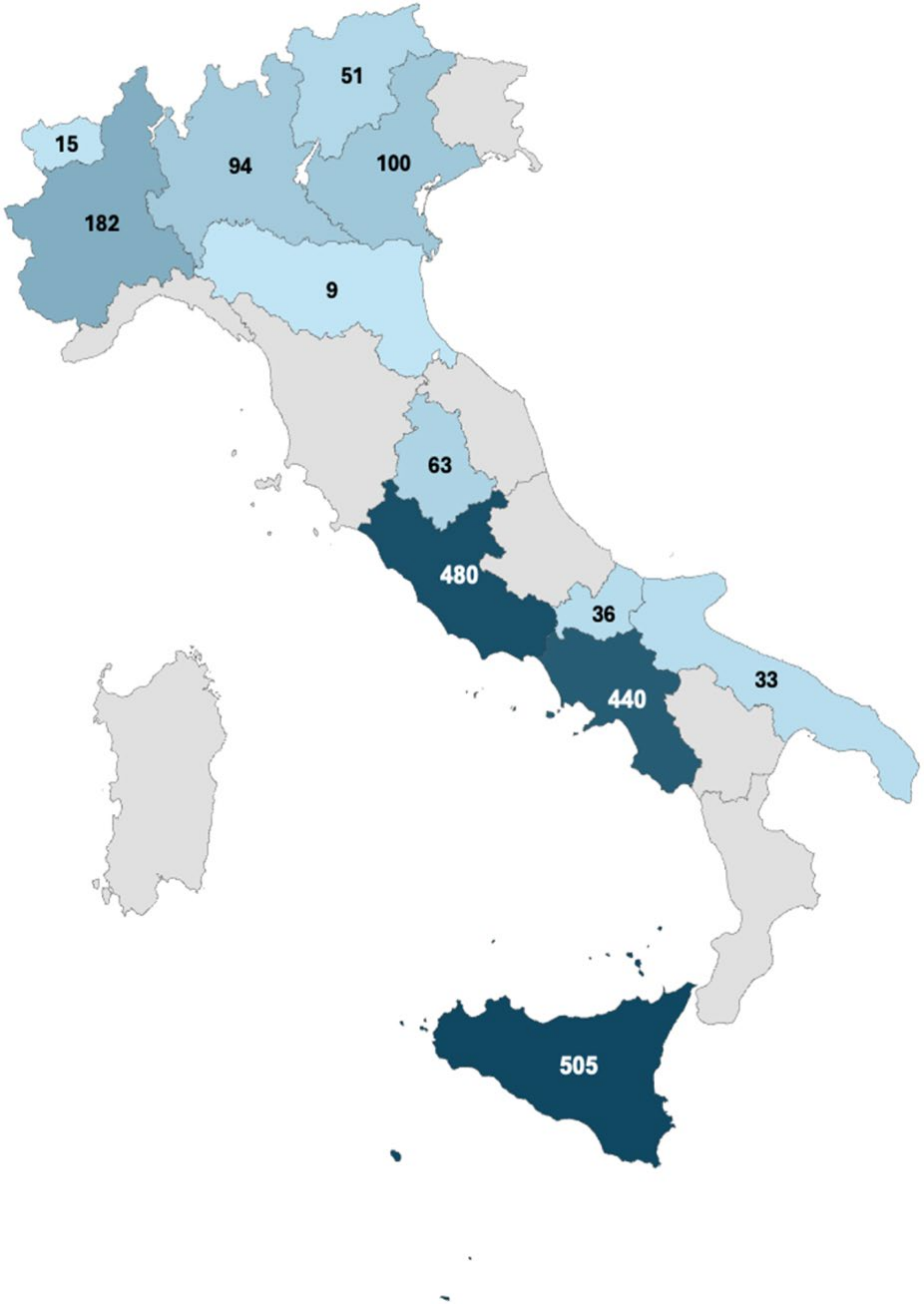
GPs trained and registered on iFDtool by region

A total of 196 licensed GPs and 1872 doctors in postgraduate training were involved in the project, showing a heterogeneous distribution of participation over the national territory, with a particular interest shown in the regions of Campania, Sicily and Lazio (Fig. 2). Thus, 90% of the participating GPs were involved through postgraduate medical schools.

The preliminary enquiry revealed an unmet educational need among Italian GPs concerning the possibility of following trainings on mental health, especially with regard to the diagnosis and treatment of patients with depressive disorder and suicide risk prevention.

A specific training program was therefore developed, divided into three distinct modules and designed to be delivered in a time frame between 2 and 3 h.

The first module of the course focused on the diagnostic criteria of major depressive disorder, referring to both ICD-11 and DSM-5. In addition, the somatic symptoms most frequently associated with depressive disorder were discussed, emphasizing the crucial role that GPs play in preventing depression. This module increased GPs’ awareness of the variety and complexity of depressive symptoms, thus facilitating an early and accurate diagnosis.

The second module focused on introducing the iFightDepression tool. This portion of the course included a practical workshop demonstrating how to use the tool and presenting the available resources for both clinicians and patients. The objective was to enhance GPs’ self-efficacy in managing depressed patients by equipping them with direct access to evidence-based and guided self-help tools.

The third and final module addressed suicide risk prevention. In this part, the role of GPs in suicide risk prevention is emphasised, dealing with suicide risk assessment and debunking false myths related to suicide. The aim is twofold: to improve GPs’ skills in detecting warning signs and to promote a proactive approach in suicide prevention.

During the 38 training courses, a keen interest emerged from the participants, who showed active participation and shared direct experiences with patients.

This dynamic transformed the courses into true opportunities for exchanging knowledge and experience, contributing significantly to the enhancement of the professional skills of the doctors involved. The training programme proposed within the iFightDeppression project represented a significant step forward in meeting the educational needs of general practitioners in Italy, with the potential to improve the quality of care and support offered to patients suffering from depressive disorders and at risk of suicide.

## Conclusion

The iFightDepression project in Italy marked a significant progress in the field of healthcare training, successfully addressing the challenges posed by the complexity of the healthcare system and the need to improve the management of depression and suicide risk among GPs. The specific training on depressive disorders and suicide risk that was developed, through the use of the iFightDepression tool, represents a replicable model that could positively influence the clinical practice of general practitioners in Italy [14, 15]. The increase of specific skills in the recognition and management of these disorders paves the way for a better quality of healthcare, with a potentially significant impact on public health and the well-being of patients [16].

The importance of understanding mental suffering also as a key factor in suicide risk assessment requires both intellectual and emotional professional effort on the part of clinicians [17, 18]. Enhancing GPs’ clinical expertise on depression is the first step towards reducing the stigma attached to mental disorder and preventing suicide risk. It emerges that the role of general practitioners is fundamental.

The iFightDepression project in Italy highlighted the need and potential of mental health training among GPs. It represents an effective model of early intervention and ongoing support for individuals suffering from depression. The dissemination and use of this tool can contribute significantly to the prevention of depression and suicidal behaviour [19], improving access to healthcare and the quality of life of patients. The training of more than 2000 doctors in Italy represents a significant step towards a more informed and proactive management of mental health.

## Data Availability

The datasets generated and/or analyzed during the current study are available from the corresponding author on reasonable request.

## Declarations

### Websites

http://www.agenas.gov.it

http://www.www.eaad.net

http://www.www.eaad-best.eu

http://www.www.gimbe.org

http://www.www.ifightdepression.com/it/

## Abbreviations

EAAD: Europear Alliance Against Depression
GPs: General Practitioners
iFD: iFightDepression
iFDt: iFightDepression tool
